# Oromucosal Alginate Films with Zein Nanoparticles as a Novel Delivery System for Digoxin

**DOI:** 10.3390/pharmaceutics13122030

**Published:** 2021-11-29

**Authors:** Daniela A. Rodrigues, Sónia P. Miguel, Jorge Loureiro, Maximiano Ribeiro, Fátima Roque, Paula Coutinho

**Affiliations:** 1 Center of Potential and Innovation in Natural Resources, Research Unit for Inland Development, Polytechnic Institute of Guarda (CPIRN-UDI/IPG), Avenida Dr. Francisco de Sá Carneiro, No. 50, 6300-559 Guarda, Portugal; danielaalmeidar@ipg.pt (D.A.R.); spmiguel@ipg.pt (S.P.M.); jcloureiro97@gmail.com (J.L.); mribeiro@ipg.pt (M.R.); froque@ipg.pt (F.R.); 2 Health Sciences Research Centre, University of Beira Interior (CICS-UBI), Avenida Infante D. Henrique, 6200-506 Covilhã, Portugal

**Keywords:** oromucosal films, sodium alginate, nanoparticle drug carriers, digoxin, zein, heart failure

## Abstract

Digoxin is a hydrophobic drug used for the treatment of heart failure that possesses a narrow therapeutic index, which raises safety concerns for toxicity. This is of utmost relevance in specific populations, such as the elderly. This study aimed to demonstrate the potential of the sodium alginate films as buccal drug delivery system containing zein nanoparticles incorporated with digoxin to reduce the number of doses, facilitating the administration with a quick onset of action. The film was prepared using the solvent casting method, whereas nanoparticles by the nanoprecipitation method. The nanoparticles incorporated with digoxin (0.25 mg/mL) exhibited a mean size of 87.20 ± 0.88 nm, a polydispersity index of 0.23 ± 0.00, and a zeta potential of 21.23 ± 0.07 mV. Digoxin was successfully encapsulated into zein nanoparticles with an encapsulation efficiency of 91% (±0.00). Films with/without glycerol and with different concentrations of ethanol were produced. The sodium alginate (SA) films with 10% ethanol demonstrated good performance for swelling (maximum of 1474%) and mechanical properties, with a mean tensile strength of 0.40 ± 0.04 MPa and an elongation at break of 27.85% (±0.58), compatible with drug delivery application into the buccal mucosa. The current study suggests that SA films with digoxin-loaded zein nanoparticles can be an effective alternative to the dosage forms available on the market for digoxin administration.

## 1. Introduction

Heart failure is a pathological condition with an estimated worldwide prevalence of 64.34 million cases representing the most significant burden after 60 years of age [1,2]. The incidence in European countries and the USA ranges from 1 to 9 cases per 1000 person-years [1]. Digoxin is a hydrophobic drug with a positive inotropic effect that reduces heart rate in supraventricular tachyarrhythmias associated with heart failure, improving the dynamic capacity of the heart [3]. It is one of the most used drugs to improve symptoms and reduce hospitalization in patients with heart failure and atrial fibrillation [4]. These conditions are highly prevalent in older adults [5]; therefore, digoxin is a widely prescribed drug at this age group [3,6]. However, this drug has a narrow therapeutic index, wide individual variability in dosage requirements, and complex metabolic pathways, which raises concern since digitalis toxicity is not only a medical emergency but can also be lethal [7]. This is of utmost relevance for the geriatric population considering the pharmacodynamic and pharmacokinetic change. The older patient’s high sensitivity to glycosides and consequent risk of intoxication [8] highlights the need of developing new delivery systems for improving safety.

Digoxin is currently only available on the market in conventional dosage forms, which are a limitation for narrow therapeutic index drugs since these have several limitations, such as reduced patient compliance, shorter half-life of drugs, and high peak, failure to control the drug release ratio, poor stability, and lack on the therapeutic target [9].

The use of mucosal administration has recently received attention by researchers on the drug administration by mucosal route since it avoids the hepatic first-pass effect and the degradation by gastrointestinal enzymes [10,11,12]. Besides that, the sublingual mucosa is very permeable with large veins, and high blood flow provides instantaneous drug absorption and high bioavailability [13]. Therefore, oral mucoadhesive drug delivery systems can be a good solution as they provide more comfort and flexibility during the administration process [14,15,16]. Moreover, mucoadhesive forms may be designed to enable prolonged retention at the site of application, providing a controlled rate of drug release for improved therapeutic outcomes [17]. Oromucosal films are alternative dosage forms to traditional solid oral dosage forms [18], which are essentially prepared through the casting method [19].

Different polymers have been used in film preparations since they achieve rapid disintegration, good mouthfeel, and mechanical properties [20] and are an easy way to enhance bioavailability [21]. In addition, the use of natural and biocompatible polymers reduces the side effects of a given drug, and biodegradable biomaterials minimizes the inflammatory effect, possesses good permeability, and good therapeutic properties [9], which overcome the toxicity and non-degradability associated with synthetic polymers used in the production of commercial dosage forms. Herein, sodium alginate (SA) was selected since it is a hydrophilic, biocompatible, and antioxidant polysaccharide [22] with mucoadhesive properties [23,24]. In fact the promising properties of SA propelled the development and commercialization of oral films for the food and pharmaceutical industries [25,26,27].

Since polymeric nanoparticles have numerous potentialities as carrier systems for bioactive compounds, it is possible to control the drug release profile and reduce drug degradation and toxicity [28,29]. Polymers are the most common materials for constructing nanoparticle-based drug carriers and among different polymers used in nanoparticle production, zein is a natural and biodegradable polymer belonging to the group of prolamins, and it is one of the few alcohol-soluble biopolymers with more than 50% hydrophobic amino acids [30]. Besides, zein has unique characteristics, such as high coating capacity, biocompatibility, low toxicity [31,32], and its mucoadhesive character can be used for mucosal delivery of drugs and vaccines [33]. This polymer was widely described as a pharmaceutical excipient in oral solid dosage forms [34]. A significant advantage of zein-based nanoparticles is their amphiphilic character, which encapsulates both hydrophilic and hydrophobic compounds like digoxin [35]. Indeed, they have been proposed to encapsulate phenolic compounds due to their ability to increase the dispersibility of drugs in an aqueous medium, as well as to promote chemical stability [36]. This polymer has been used in modified release systems for the delivery of enzymes, drugs, essential oils, and other substances [32,33]. In fact, different studies have reported the ability of zein nanoparticles to encapsulate different drugs, such as lovastatin [37], artemether [38], gambogenic acid [39], doxorubicin [40], 5-fluorouracil [41], docetaxel [42], carvacrol [43], and maytansine [44]. These studies demonstrated the utility of zein nanoparticles as a viable drug-delivery and in a recent work, PEG-coated zein nanoparticles demonstrated to be adequate carriers for promoting the oral bioavailability of biomacromolecules [45].

Herein, the zein nanoparticles were produced through the nanoprecipitation technique, also known as solvent displacement, or antisolvent method, which consists of the interfacial interaction of zein after displacement of a semi-polar solvent, miscible in water. The rapid diffusion of the organic solvent results in the reduction of the interfacial tension between the two phases, increasing the surface area, inducing supersaturation, leading to precipitation of the solute and the formation of nanoparticles [46]. This method employs the addition of zein solution to an anti-solvent (water), which allows the controlled protein precipitation due to the reduction of solubility in the medium, promoting the nanoparticles’ formation.

However, it is important to notice that the physicochemical properties of the nanoparticles (e.g., size, surface properties, and polydispersity index (PDI)) are dependent on the materials and technique used for the nanoparticles production. In this case, the production of zein nanoparticles through the nanoprecipitation technique enables the production of the reproducible and positively charged zein nanoparticles that will promote electrostatic interactions with negatively sialic acid residues in mucin, which will prolong the residence time in the buccal mucosa and consequently increase the drug bioavailability [47,48,49].

In turn, the oromucosal films will act as a matrix to support the incorporation of the digoxin-loaded zein nanoparticles since they are flexible, comfortable, and easy to administer, prolonging the stability of the system [50]. Herein, this study aimed to develop a sodium-alginate mucoadhesive film containing zein nanoparticles with embedded digoxin to be used as a buccal drug delivery system. The films were produced using the casting method from aqueous solutions and the nanoparticles were obtained through the nanoprecipitation method.

## 2. Materials and Methods

### 2.1. Materials

Standard digoxin 96% was purchased from Alfa Aesar (Haverhill, MA, USA). SA (molecular weight 10,000–600,000 g/mol) was obtained from AppliChem GmbH (Darmstadt, Germany). Glycerol was acquired from Guinama S.L.U (Valencia, Spain. Ethanol 100% was purchased from Carlo Erba Reagents (Cornaredo, Italy) with a density at 20 °C of 0.7893:0.7899. Zein was obtained from Acros Organics (Waltham, MA, USA). Sodium chloride 99.5% was obtained from Honeywell Fluka (Charlotte, NC, USA), potassium phosphate monobasic and sodium phosphate dibasic and High-Performance Liquid Chromatography (HPLC)-gradient grade acetonitrile 99.9% were obtained from VWR Chemicals (Radnor, PA, USA). HPLC-gradient grade methanol 99.9% was obtained from Chem-Lab NV (Zedelgem, Belgium). Deionized water was used for all sample preparation.

### 2.2. Preparation of Sodium Alginate Films

Films were prepared using the casting method from aqueous solutions, as previously reported [51]. An aqueous solution of SA (3% *w*/*v*), was placed under magnetic stirring at 25 °C and 400 rpm, for 6 h. After that, the ethanol was added at different concentrations (0, 10 and 20% *v*/*v*) to promote the gelation of the film [51]. Then, the glycerol (12 g/L) was added as a plasticizer for the film optimization due to its texture and mechanical properties [52]. The polymeric solution was deposited into Petri plates (55 mm) and was dried at 30 °C overnight (Incubator Hood TH 15-Edmund, Bühler GmbH, Uzwill, Switzerland). The final composition of the oromucosal films is shown in Table 1. Posteriorly, SA films with embedded zein-digoxin nanoparticles were also produced. The production process of nanoparticles is described in Section 2.4.

### 2.3. Characterization of Films

#### 2.3.1. Scanning Electron Microscopy Analysis

The morphology and structure of the surface films were characterized through scanning electron microscopy analysis. The samples were mounted onto aluminum stubs with Araldite glue and sputter-coated with gold using a Quorum Q150R ES sputter coater (Quorum Technologies Ltd., Laughton, Lewes, UK). Then the images acquired with different magnifications were acquired in a Hitachi S-3400N Scanning Electron Microscope (Hitachi, Tokyo, Japan) at an acceleration voltage of 20 kV.

#### 2.3.2. Fourier Transform Infrared Spectroscopy (FTIR)

Fourier Transform Infrared Spectroscopy (FTIR) measurements were performed to evaluate the effect of ethanol addition in SA structure by a Nicolet iS10 FTIR spectrophotometer (Thermo Scientific, Waltham, MA, USA). The analysis was performed with an average of 128 scans, a spectral width ranging from 4000 and 400 cm^−1^, and a spectral resolution of 4 cm^−1^. At least three replicates were run for each sample.

#### 2.3.3. Thickness

The film thickness was measured using a Digimatic Caliper (0.01 mm, Mitutoyo Corporation, Sakado, Japan) at 10 different film positions. At least three replicates for each formulation were considered.

#### 2.3.4. Mechanical Properties

Tensile strength (TS) and elongation at break (EAB) were measured using a Texture Analyser (TA-XT Plus, Stable Micro Systems, Godalming, UK). All measurements were performed in three replicates for each formulation. Each test strip was cut into a specific size (3 × 1 × 0.1 cm) and placed longitudinally in a tensile grip probe (A/MTG). Initial grip separation was 5 mm and crosshead speed was 10 mm/min. The test was considered concluded at the film break. The TS was evaluated using the Equation below (1). Results were expressed in MPa.

(1) $$\;\mathrm{TS}=\frac{\mathrm{Force}\;\mathrm{at}\;\mathrm{break}}{\mathrm{Thickness}\times \mathrm{width}}$$

The EAB was calculated according to the following Equation (2):

(2) $$\;\mathrm{EAB}\;\left(\%\right)=\frac{\mathrm{Increase}\;\mathrm{in}\;\mathrm{length}}{\mathrm{Original}\;\mathrm{length}}\times 100$$

#### 2.3.5. Swelling Profile

The swelling profile of the films was measured by a method previously proposed [51]. The samples (2 × 2 cm) were immersed in 1 mL of a simulated saliva solution prepared with sodium chloride (8.00 g/L), potassium phosphate monobasic (0.19 g/L), and sodium phosphate dibasic (2.38 g/L), setting pH to 6.8 [53], at 25 °C and stirred at 120 rpm for 5 min. The samples were evaluated after 0, 30, 60, 120, 180, 240, and 300 s of the beginning of the test. The excess of simulated saliva solution was removed with a filter paper and their wet weight was immediately determined to calculate the swelling ratio by the following Equation (3), where W_t_ is the final weight, and W_0_ is the initial weight of the films:

(3) $$\;\mathrm{Swelling}\;\left(\%\right)=\frac{W_{t}}{W_{0}}\times 100$$

#### 2.3.6. Dissolution Time

To investigate the dissolution time of the films, the samples (1 × 1 cm) were immersed in 5 mL of simulated saliva solution at 37 °C and at 120 rpm. The test was considered concluded when the film was completely dissolved. Measurements were performed in triplicate for each formulation.

### 2.4. Preparation of Zein Nanoparticles with Embedded Digoxin

Zein (2.5 mg/mL) was dissolved in ethanol (80% *v*/*v*). Nanoparticles were prepared by the nanoprecipitation method through confined impingement jets with dilution (CIJ-D) mixer, as previously described [54]. CIJ-D mixer is made of a high-density polyethylene, with two inlets and adapters fitted with threaded syringes, and one outlet adapter. The dimensions and operating mode is described in more detail in work conducted by Han et al. [54]. One of the syringes contains 2.50 mL zein solution and digoxin at different concentrations (0.00, 0.25, 0.50, and 1.00 mg/mL), and the second syringe contains 2.50 mL of deionized water. Nanoparticles without digoxin were also produced as a control. The two syringes were then attached to the two vertical openings on the CIJ-D mixer. A beaker containing 45 mL of deionized water was placed at the exit of the CIJ-D mixer. The exit stream outlet was submerged in the water. The two syringes were then pushed rapidly and simultaneously by hand to inject the liquids into the CIJ-D mixer at equal rates, where the two streams were rapidly mixed and collected in water solution.

### 2.5. Particle Size, Zeta Potential and Polydispersity Index (PDI)

After production, zein nanoparticles with and without digoxin, size distribution, zeta potential, and PDI were determined by dynamic scattering technique using Zetasizer Nano ZS, Malvern Instruments, Malvern, UK. Such parameters were also measured over time, for 26 days, at room temperature, to evaluate the stability of the nanoparticles.

### 2.6. Determination of Standard Calibration Curve and Encapsulation Efficiency of Digoxin into Nanoparticles

Encapsulation efficiency (EE) of digoxin was determined by HPLC. A standard calibration curve was previously obtained (y = 292.67 x + 17.595, R^2^ = 0.999). HPLC chromatography was performed according to the conditions described previously [55], with a column C18 (Acclaim™ 120 Reversed-Phase Columns C18, 5 μm, 4.6 × 150 mm, Thermo Scientific) at a temperature of 35 °C and the mobile phase (mixture of water and acetonitrile, 72:28% (*v*/*v*)) was pumped at a flow rate of 0.8 mL/min. The run time cycle was completed in 20 min. Peak areas registered at 218 nm were used for digoxin quantification. All experiments were carried out in triplicate.

The EE indicates the drug amount into nanoparticles and was determined after ultrafiltration-centrifugation (Amicon^®^ Ultra Centrifugal Filters, 30k; Merck Millipore, Billerica, MA, USA). The filtrate, containing the unencapsulated drug, which can pass through the filter membrane during centrifugation (4000× *g*; 10 min), was analyzed by HPLC. Encapsulation efficiency was calculated using the following Equation (4):

(4) $$\;\mathrm{EE}\;\left(\%\right)=\frac{\mathrm{Actual}\;\mathrm{amount}\;\mathrm{of}\;\mathrm{drug}-\mathrm{loaded}\;\mathrm{in}\;\mathrm{nanoparticles}}{\mathrm{Theory}\;\mathrm{amount}\;\mathrm{of}\;\mathrm{drug}-\mathrm{loaded}\;\mathrm{in}\;\mathrm{nanoparticles}}\times 100$$

### 2.7. Statistical Analysis

The statistical analysis of the obtained results was performed using one-way analysis of variance (ANOVA), with Tukey’s test for post hoc analysis, using GraphPad Prism software version 8.0.1 (Dr Harvey Motulsky, San Diego, CA, USA). All of the results were expressed as the mean value ± standard error of the mean (SEM). A *p*-value lower than 0.05 (*p* < 0.05) was considered statistically significant.

## 3. Results and Discussion

### 3.1. Scanning Electron Microscopy Analysis

Scanning electron microscopy analysis was performed to characterize the film’s surface, as previously reported in other studies [56,57]. The images indicate that the morphology of all selected films is dense, homogeneous, and has no pores and cracks (Figure S1). The addition of glycerol and ethanol did not change the surface of the developed films. These characteristics, namely the absence of pores and surface uniformity, represent good film quality and are appropriate for buccal drug delivery systems [19,58]. In fact, other authors also state that the oral films must be homogeneous and smooth, not presenting with bubbles, cracks, or aggregates, which aims to improve its acceptability [59].

### 3.2. Fourier Transform Infrared Spectroscopy (FTIR) Analysis

**Figure 1:**
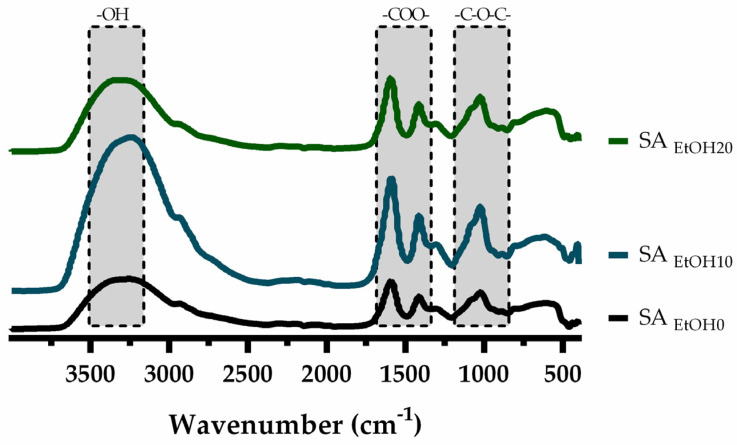
FTIR spectra (SA: Sodium alginate, EtOH: Ethanol).

FTIR spectra of SA films are presented in Figure 1. For native SA film, the peaks at 3253 cm^−1^ and 1023 cm^−1^ were assigned to stretching vibrations of –OH and –C–O–C– bonds and asymmetric and symmetric –COO– stretching at 1590 cm^−1^ and 1413 cm^−1^, respectively [51,60,61]. In this work, ethanol was used for gelation of SA chains. Although ionic crosslinking is commonly used in SA films, another study observed that when the SA films were crosslinked with Ca^2+^ ions, the peaks become broader. Thus, different non-conventional crosslinking methods have been used, such as the non-solvent method, which commonly uses ethanol. In fact, it has been described that the gelation of polysaccharides induced by ethanol can occur due to the low water activity, wherein the polysaccharide-water interactions are minimized and the hydrophobic interactions between polysaccharide chains are promoted [62,63]. Since the gelation occurred directly in ethanol there was no need for the solvent-exchange step and the process occurred without additional crosslinkers.

It is possible to verify that both the asymmetric and symmetric –COO– peaks of the films did not change with increasing the ethanol proportion. Thus, ethanol fulfilled its purpose without changing the SA structure, as seen in another study [51].

### 3.3. Thickness

**Figure 2:**
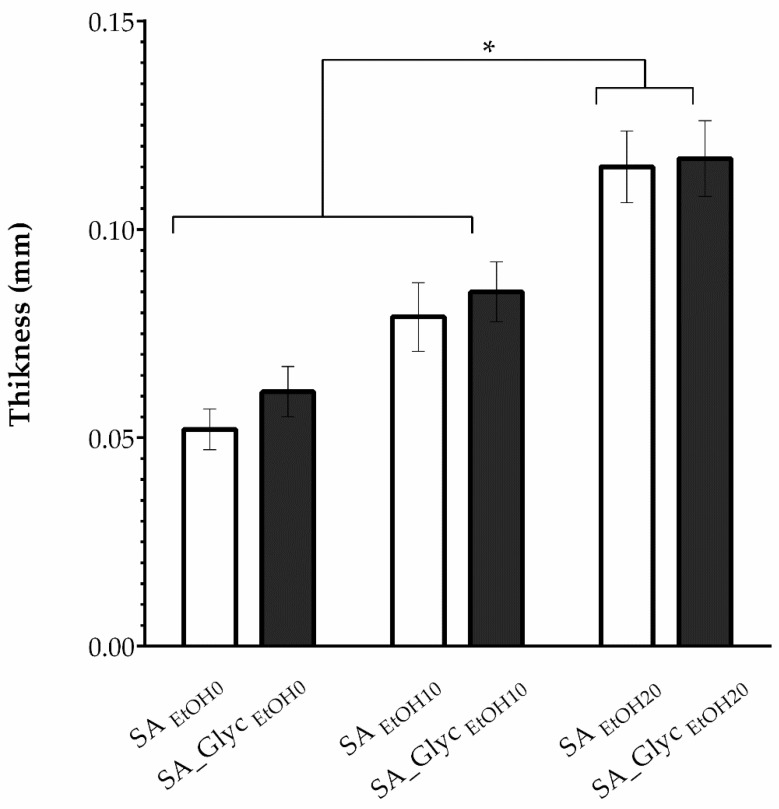
Thickness of the films with and without glycerol considering the gelling agent (ethanol) (SA: Sodium alginate, EtOH: Ethanol, and Glyc: Glycerol, * *p* < 0.05).

Apart from ethanol, the SA films were also composed of glycerol. Such a plasticizer has been commonly used since it improves the flexibility of films [64]. The thickness of SA films changed depending on the ethanol content and glycerol addition, as shown in Figure 2. The addition of ethanol changed the thickness of films leading to thicker films. This may be related to the structural modification in the polypeptide chain that the gelation process imposes since the three-dimensional structure assumes a conformation that takes up more space and, therefore, the greater thickness of the reticulated films [65]. It was possible to verify that the addition of glycerol does not affect film thickness (Figure 2). Despite some minor changes on the films’ thickness, all formulations presented values within the suitable range (0.05–1 mm), which is considered ideal to reduce side effects and extensive metabolism of drugs in buccal films [66]. In turn, the thickness values maintain along the surface of the film, assuring the thickness uniformity, which is important for film dose accuracy [19].

### 3.4. Mechanical Properties

TS is defined as a measurement of the maximum amount of force applied at which the film breaks and is used to characterize the mechanical strength of the films [67]. In turn, the percentage increase in EAB is the length that a material can be extended/stretched before it breaks. It is related to the elasticity of the material and the ability of a plastic specimen to resist changes of shape without cracking. An ideal oromucosal film dosage form should be flexible, elastic, and soft, but strong enough to resist breaking caused by stress from mouth movements [13].

**Figure 3:**
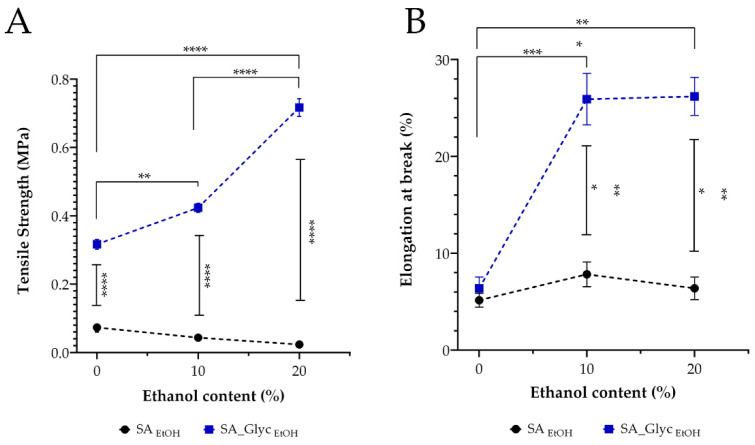
Mechanical properties of the films (*n* = 3). (**A**)**:** Tensile strength. (**B**)**:** Elongation at break. * *p* < 0.05, ** *p* < 0.1; *** *p* < 0.01; **** *p* <0.0001.

Through the analysis of results presented in Table 2, it is possible to notice that the addition of glycerol promoted a decrease in TS values and an increase in EAB. It is widely reported that the plasticizers interfere with polymer chains, promoting a decrease in intermolecular forces, soften the rigidity of the film’s structure, and increase the polymer mobility. Thus, the presence of glycerol leads to a ductile and flexible material [64]. The increase in ethanol led to an increase in the TS and a decrease in EAB values, except for films without glycerol (Figure 3). A significant decrease in TS was noticed in another study for gelatin films incorporated with the highest curcuma ethanol extract content [68]. The increase in the EAB values can be explained by the good physical gelation process between the polymeric matrix and the incorporated ethanol, leading to more cohesive and flexible matrices [68].

### 3.5. Swelling Profile

**Figure 4:**
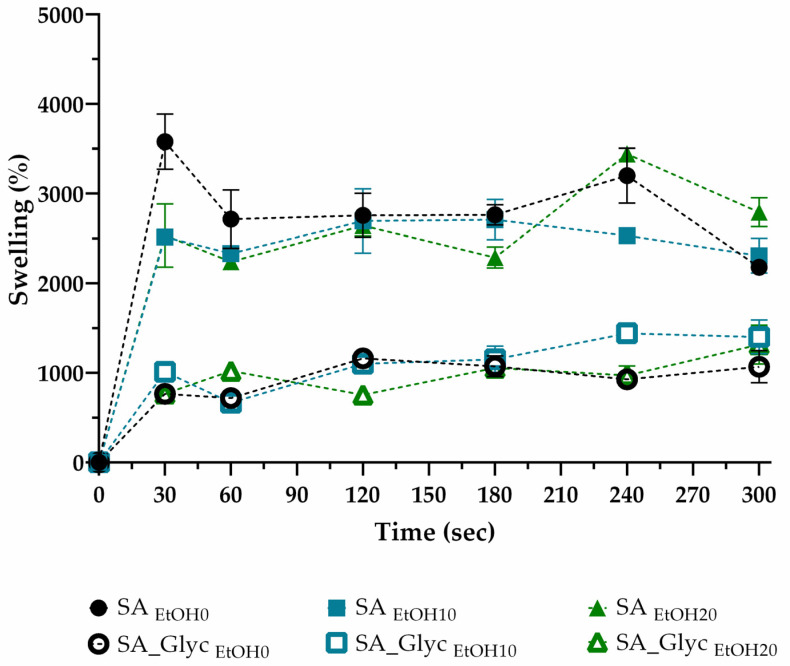
Swelling profile of the produced films (SA: Sodium alginate, EtOH: Ethanol, and Glyc: Glycerol).

Polymeric film swelling is important to understand films’ water absorption capacity and obtain information about their water resistance [69]. The swelling profile of SA films, with and without glycerol treated with different ethanol concentrations, was recorded in Figure 4. The data obtained revealed the high-water absorption ability of the films in the first 30 s, stabilizing the swelling behavior over at least 300 s. This profile is described as suitable for buccal administration since polymers with high initial swelling rate could promote mucoadhesion [70]. The graphics also showed that after 240 s, the degradation of the film occurs for formulations without glycerol. In general, all glycerol-free films exhibit similar behavior with a maximum swelling percentage of 3578.7% ± 308.23.

In contrast, glycerol promoted a more controlled swelling profile in all formulations where the maximum swelling was 1441% ± 4.041. Adding a hydrophobic compound, such as glycerol, will impair the interaction with water molecules, decreasing the water absorption capacity. Another study reported that the increase in glycerol concentration reduced the swelling index of the fast oral dissolving films studied [71]. The swelling capacity of SA films is facilitated by carboxylic groups, which are strongly associated with water molecules [65]. In a drug delivery system, a moderate swelling profile is desirable, not compromising the system’s stability [72]. Thus, based on the results, the SA_Glyc _EtOH10_ formulation was selected to incorporate zein nanoparticles since it presented a swelling profile compatible to be applied as mucoadhesive drug delivery system for the buccal mucosa.

### 3.6. Dissolution Time

The results obtained (Table 3) showed that the presence of ethanol induces an increase on dissolution time, which can be explained by the reduction of water molecule interactions and consequently delay the dissolution time [73]. In terms of the glycerol addition, the effects were just observed on formulations with 10% of ethanol, which increased the dissolution time [71].

### 3.7. Characterization of Zein Nanoparticles

Nanoparticle drug carriers aim to achieve more efficient and sustained drug delivery. Their characteristics, such as particle size, charge, and hydrophobicity, are determinants of the permeability of the mucosal barrier [74]. The mean size of the zein nanoparticles without digoxin was 85.72 ± 0.360 nm, which increase when the digoxin concentration also augmented (Table 4). However, all produced nanoparticles displayed a mean diameter inferior to 200 nm, which is ideal for escaping recognition by the reticuloendothelial system and, consequently, prolong their half-life in the blood system [74]. Nevertheless, other authors described the particle size reduction of nanosuspensions as determinant for the increase in the surface area and enhancement in dissolution velocity of the drug [75,76]. In all cases, the PDI of the formulations remained under 0.4, indicating monodisperse nanosuspensions. In turn, the zeta potential indicates the surface charge of nanoparticles presenting values above 20 mV, considered as moderately stable [77,78,79]. Apart from the size of nanoparticles, their uptake is also dependent on charge density. The positive charge of zein nanoparticles and hydrophobic character of zein nanoparticles enables the ionic interaction with negatively charged groups available on the cell membrane surface and improves the epithelial endocytosis through the attachment of polymeric substances to the glycoproteins on epithelial surfaces, which allows for the increase of the mucoadhesion phenomenon [80,81,82]. This provides an intimate contact between drug formulation and mucosa tissue, increasing the drug absorption and residence time, resulting in improved drug therapeutic activity through high drug flux at absorptive mucosa [83]. Moreover, the mucoadhesive adhesion of zein nanoparticles on porcine buccal mucosa was already evaluated by other authors, who verified that the positively charged zein nanoparticles can anchor to the mucus layer by electrostatic interactions with negatively charged sialic acid residues in mucin, which is fundamental to prolong the residence time in the buccal mucosa [84].

**Figure 5:**
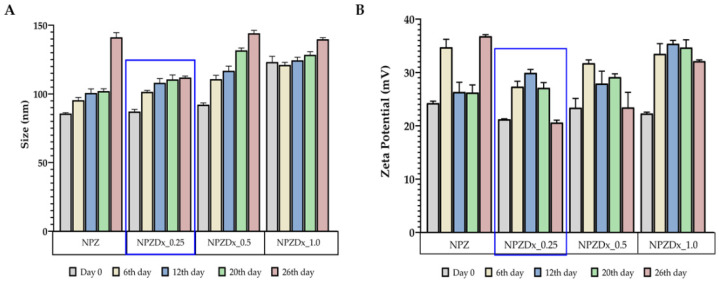
Stability of nanoparticles over time, *n* = 3 (NPZ: Zein nanoparticles, NPZDx: Zein-digoxin nanoparticles): (**A**)**:** Particle size (nm). (**B**)**:** Zeta potential (mV). The blue square evidence the nanoparticles formulation with more suitable morphological properties.

In addition, the stability of the aqueous nanoparticles suspension, stored at room temperature, was evaluated for 26 days (Figure 5). It was possible to verify that the zein nanoparticles incorporating 0.25 mg/mL of digoxin maintained their size for 12 days, with a PDI value of 0.23 ± 0.00, suggesting an ideal stability. In terms of zeta potential, no significant differences were observed between 6 and 20 days (*p* > 0.999) for this formulation. Thus, it can be concluded that this formulation is stable over at least 20 days and it was chosen to be incorporated into the film. Through this strategy, it is possible to combine the mucoadhesive behavior of zein, allowing the strong electrostatic interactions with mucin, as highlighted previously [84], and acting as novel platform for the buccal delivery of the poorly water-soluble digoxin.

### 3.8. Digoxin Encapsulation Efficiency

The digoxin was incorporated into zein nanoparticles with a drug encapsulation efficiency of 91 ± 0.03%. This result is of utmost relevance since high drug encapsulation allows for lower concentrations of nanoparticles compared to other dosage forms, and the controlled delivery of drugs. To the best of our knowledge, this is the first work evaluating the performance of zein nanoparticles for digoxin delivery. In other studies it is only possible to find works reporting the production of zein nanoparticles incorporated with other biologically active compounds, achieving encapsulation efficiencies ranging between 47.80 and 92.60% [40,85,86,87]. One study reported the use of simple coacervates of zein to encapsulate gitoxin, a naturally occurring cardiac glycoside (such as digoxin) and proved the stability and maintenance of the biological activity of glycosides [88]. On the other hand, the incorporation of digoxin into other polymeric nanoparticles has been previously described, and the results showed high encapsulation efficiency and revealed that digoxin-loaded nanoparticles increased permeability across cell layers [89]. In addition, the digoxin incorporation into nanosystems affords different potentialities, as verified by Das et al., where the in vivo studies revealed an increase on digoxin bioavailability in solid lipid nanoparticles when compared with a digoxin solution after oral administration (0.25 mg) [90]. Taking this into account, we consider that the digoxin encapsulation into zein nanoparticles should not compromise its biological activity and represents a promising approach for the development of novel and safer formulations.

### 3.9. Characterization of SA Films Embedded with Zein-Digoxin Nanoparticles

Considering the optimal film formulation obtained, the nanoparticles suspension was embedded into the formulation by direct mixing with the SA_glycerol solution. The casting method with ethanol for film production followed the previous described approach. The results obtained for the characterization of films embedded with zein-digoxin (ZnDx) nanoparticles are summarized in Table 5. The addition of nanoparticles into the film did not change their thickness, with a mean value of 0.08 ± 0.90 mm. In turn, a significant decrease of EAB of the film (5.72 ± 0.58) was registered, while TS was not changed. Such can occur due to the addition of hydrophobic compounds, such as zein and digoxin, which prevent the interaction between the hydrophilic groups of SA and water [91].

**Figure 6:**
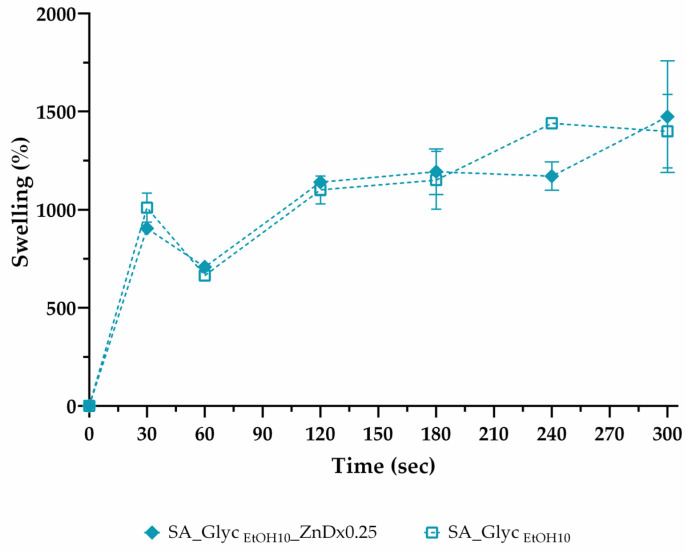
Swelling profile of the optimized film with zein-digoxin nanoparticles incorporated (SA: Sodium alginate, EtOH: Ethanol, Glyc: Glycerol, Zn: Zein, and Dx: Digoxin). Results are expressed as mean ± SEM.

In terms of the swelling profile, there are no significant differences for both formulations (Figure 6). These formulations demonstrated an increase in the swelling capability after 30 s, suggesting a suitable film swelling, able to accelerate the release of the drug by diffusion and erosion [92]. Drug-loaded films dissolved significantly slower (*p* = 0.02) than the equivalent drug-free formulation (Table 5). This could be due to the poorly water-soluble character of digoxin. Besides, hydrophobic polymers (zein) do not allow for quick hydration upon contact with simulated saliva and, hence, delay the dissolution time of the films [93].

## 4. Conclusions

In this study, a novel SA_Glyc _EtOH10_ film containing ZnD × 0.25 nanoparticles was successfully prepared by incorporating drug nanosuspensions with mucoadhesive films mainly composed of SA, which provides a new route of transforming nanosuspensions of poorly water-soluble drugs (such as digoxin) into a solid dosage, which due to the bioadhesive behavior, reduced the number of administrations. SA films were successfully prepared by solution casting with different concentrations of ethanol. Then, the effect of the glycerol plasticizing agent on films’ properties was also evaluated, where it was verified that the films containing glycerol presented a more controlled swelling profile. In addition, the zein nanoparticles incorporating digoxin were also successfully produced through the nanoprecipitation method, which displayed a size and surface charge stable for at least 12 days.

In this way, the mucoadhesive SA film incorporated with ZnD × 0.25 nanoparticles presented a swelling profile and mechanical properties compatible with the application as drug delivery system through the buccal mucosa. The development of this technological innovation becomes pertinent and necessary since it allows for the achievement of a more controlled drug release, greater therapeutic effect, reduction of side effects, and also to improve therapeutic compliance in patients with dysphagia.

In the near future, complementary assays, such as drug permeation, mucoadhesiveness, pharmacokinetic profile, and bioavailability, would allow for the successful scale-up of the new oromucosal film produced as alternative dosage form for digoxin and for drugs suffering from first-pass effect, especially those with a narrow therapeutic index.

## Figures and Tables

**Table 1:** Film’s composition (SA: Sodium alginate, EtOH: Ethanol, and Glyc: Glycerol).

Formulation	Film Composition			
	3% SA Solution (mL)	Ethanol (mL)	Water (mL)	Glycerol (g/L)
SA _EtOH0_	15.00	0.00	0.00	0.00
SA _EtOH10_	10.00	1.50	3.50	0.00
SA _EtOH20_	10.00	3.00	2.00	0.00
SA_Glyc _EtOH0_	15.00	0.00	0.00	12.00
SA_Glyc _EtOH10_	10.00	1.50	3.50	12.00
SA_Glyc _EtOH20_	10.00	3.00	2.00	12.00

**Table 2:** The mechanical characterization of oromucosal films (*n* = 3) is expressed as mean ± SEM (SA: Sodium alginate, EtOH: Ethanol, and Glyc: Glycerol).

Formulation	Tensile Strength (MPa)	Elongation at Break (%)
SA _EtOH0_	0.07 ± 0.01	5.15 ± 0.70
SA_Glyc _EtOH0_	0.35 ± 0.02	41.97 ± 0.72
SA _EtOH10_	0.04 ± 0.00	7.83 ± 1.27
SA_Glyc _EtOH10_	0.42 ± 0.01	27.85 ± 4.59
SA _EtOH20_	0.02 ± 0.00	6.38 ± 1.17
SA_Glyc _EtOH20_	0.72 ± 0.03	26.19 ± 1.96

**Table 3:** The dissolution time of oromucosal films (*n* = 3) is expressed as mean ± SEM (SA: Sodium alginate, EtOH: Ethanol, and Glyc: Glycerol).

Formulation	Dissolution Time (min)
SA _EtOH0_	7.10 ± 0.41
SA_Glyc _EtOH0_	6.27 ± 0.18
SA _EtOH10_	8.88 ± 0.04
SA_Glyc _EtOH10_	11.36 ± 0.68
SA _EtOH20_	10.38 ± 0.05
SA_Glyc _EtOH20_	9.00 ± 0.65

**Table 4:** Characterization of the nanoparticles according to the digoxin concentration (*n* = 3), data are expressed as mean ± SEM (PDI: Polydispersity Index).

Formulation		Mean Size (nm)	PDI	Zeta Potential (mV)
Zein 2.5 mg/mL	Digoxin 0.00 mg/mL	85.72 ± 0.36	0.22 ± 0.00	24.23 ± 0.39
	Digoxin 0.25 mg/mL	87.20 ± 0.88	0.23 ± 0.00	21.23 ± 0.07
	Digoxin 0.50 mg/mL	92.16 ± 0.77	0.20 ± 0.01	23.40 ± 1.72
	Digoxin 1.00 mg/mL	123.20 ± 2.42	0.36 ± 0.01	22.30 ± 0.25

**Table 5:** Characterization of the final formulation, data are expressed as mean ± SEM (SA: Sodium alginate, Glyc: Glycerol, EtOH: Ethanol, Zn: Zein, and Dx: Digoxin).

Formulation	Thickness (mm)	Tensile Strength (MPa)	Elongation at Break (%)	Dissolution Time (min)
SA_Glyc _EtOH10_	0.09 ± 0.02	0.42 ± 0.01	27.85 ± 4.59	11.36 ± 0.68
SA_Glyc _EtOH10__ZnDx0.25	0.08 ± 0.90	0.40 ± 0.04	5.72 ± 0.58	13.75 ± 0.37

## Data Availability

Not applicable.

## Supplementary Materials

The following are available online at https://www.mdpi.com/article/10.3390/pharmaceutics13122030/s1, Figure S1: SEM micrographs of films morphology (SA: Sodium alginate, EtOH: Ethanol, and Glyc: Glycerol). A: SA _EtOH0_. B: SA _EtOH10_. C: SA_Glyc _EtOH0_. D: SA_Glyc _EtOH10_.
