# Supplementary material for: Oromucosal Alginate Films with Zein Nanoparticles as a Novel Delivery System for Digoxin
Source: Pharmaceutics. 2021 Nov 29;13(12):2030. doi: 10.3390/pharmaceutics13122030 (PMC8706652; doi:10.3390/pharmaceutics13122030)
Supplement: Supplementary file 1 [file pharmaceutics-13-02030-s001.zip › pharmaceutics-1429392-supplementary.pdf]

pharmaceutics

Supplementary Materials: Oromucosal Alginate Films with Zein Nanoparticles as a Novel Delivery System for Digoxin

Daniela A. Rodrigues, Sónia P. Miguel, Jorge Loureiro, Maximiano Ribeiro, Fátima Roque and Paula Coutinho

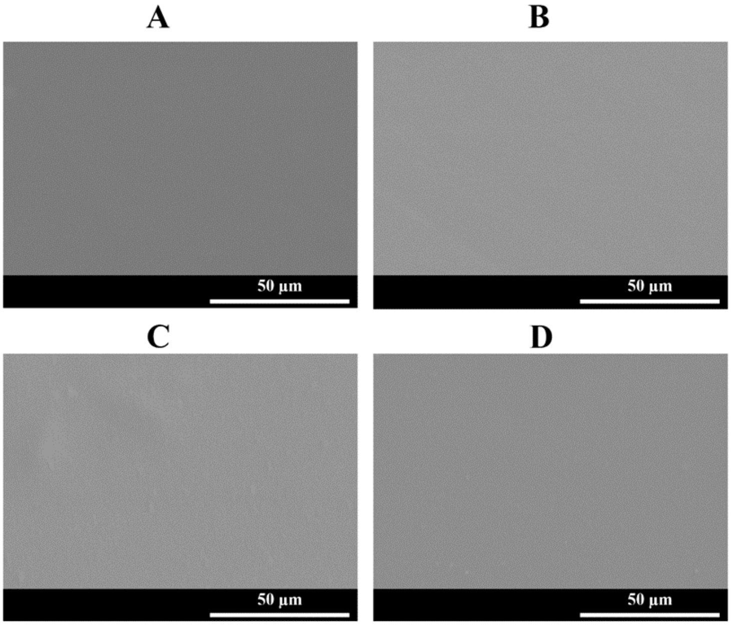

Figure S1. SEM micrographs of films morphology (SA: Sodium alginate, EtOH: Ethanol, Glyc: Glycerol). (**A**) SA_EtOH0. (**B**) SA_EtOH10. (**C**) SA_Glyc_EtOH0. (**D**) SA_Glyc_EtOH10.
